# 

**Published:** 1807-07

**Authors:** 


### MEDICAL AND PHYSICAL

### JOURNAL.

THE

### MEDICAL

AND

### PHYSICAL

### *JOURNAL.*

CONDUCTED BY

T. BRADLEY, M.D.

AND

R. BATTY, M.D.

VOL. XVIII.

FROM JUNE TO DECEMBER, 1807.

*LONDON:*

PRINTED FOR RICHARD PHILLIPS, NO. 6, BRIDGE-STREET, BLACKFRIARS,

By William Thorne, Red Lion Court, Fleet Street.

### NEW MEDICAL PUBLICATIONS.

The Code of Health and Longevity ; or a concise View of the Principles calculated for the Preservation of Health, and the Attainment of long Life; by Sir John Sinclair, Bart. 4 vols. 8vo. 48s. boards.

A short System of Comparative Anatomy ; by J. F. Blumenbach. 8vo. 12s. boards.

The Anatomy and Surgical Treatment of Crural and Umbilical Hernia, &c. &c. by Astley Cooper. Part II. Atlas folio, 42s. boards.

The Cause of the Yellow Fever, and the Means of preventing it in places not yet infected with it; by Thomas Paine. 1s.

A Description of Medicine Chests, with their Contents, as adapted to different Climates; to which are added a Catalogue of Drugs, &c. &c. 2s

### To CORRESPONDENTS.

Communications are received from Dr. Jardine, Mr. Scammell, Mr. Smyth, Mr. Jenkinson, Dr. Bellamy, Dr. Edin, Mr. Ring, C. H. W. and A. F.

